# Identification of Larvicidal Constituents of the Essential Oil of *Echinops grijsii* Roots against the Three Species of Mosquitoes

**DOI:** 10.3390/molecules22020205

**Published:** 2017-01-27

**Authors:** Mei Ping Zhao, Qi Zhi Liu, Qiyong Liu, Zhi Long Liu

**Affiliations:** 1 Department of Entomology, China Agricultural University, Haidian District, Beijing 100193, China; zhaomeiping@ioz.ac.cn (M.P.Z.); lqzwyz@cau.edu.cn (Q.Z.L.); 2 State Key Laboratory of Infectious Disease Prevention and Control, Collaborative Innovation Center of Diagnosis and Treatment of Infectious Diseases, National Institute for Communicable Disease Control and Prevention, Chinese Center for Disease Control and Prevention, Beijing 102206, China; liuqiyong@icdc.cn

**Keywords:** *Echinops grijsii*, *Aedes albopictus*, *Anopheles sinensis*, *Culex pipiens pallens*, larvicidal activity

## Abstract

The screening of Chinese medicinal herbs for insecticidal principles showed that the essential oil of *Echinops grijsii* Hance roots possessed significant larvicidal activity against mosquitoes. The essential oil was extracted via hydrodistillation and its constituents were determined by gas chromatography-mass spectrometry (GC-MS) analysis. GC-MS analyses revealed the presence of 31 components, with 5-(3-buten-1-yn-1-yl)-2,2′-bithiophene (5-BBT, 27.63%), α-terthienyl (α-T, 14.95%), 1,8-cineole (5.56%) and *cis*-β-ocimene (5.01%) being the four major constituents. Based bioactivity-directed chromatographic separation of the essential oil led to the isolation of 5-BBT, 5-(4-isovaleroyloxybut-1-ynyl)-2,2′-bithiophene (5-IBT) and α-T as active compounds. The essential oil of *E. grijsii* exhibited larvicidal activity against the fourth instar larvae of *Aedes albopictus*, *Anopheles sinensis* and *Culex pipiens pallens* with LC_50_ values of 2.65 μg/mL, 3.43 μg/mL and 1.47 μg/mL, respectively. The isolated thiophenes, 5-BBT and 5-IBT, possessed strong larvicidal activity against the fourth instar larvae of *Ae. albopictus* (LC_50_ = 0.34 μg/mL and 0.45 μg/mL, respectively) and *An. sinensis* (LC_50_ = 1.36 μg/mL and 5.36 μg/mL, respectively). The two isolated thiophenes also had LC_50_ values against the fourth instar larvae of *C. pipiens pallens* of 0.12 μg/mL and 0.33 μg/mL, respectively. The findings indicated that the essential oil of *E. grijsii* roots and the isolated thiophenes have an excellent potential for use in the control of *Ae. albopictus*, *An. sinensis* and *C. pipiens pallens* larvae and could be used in the search for new, safer and more effective natural compounds as larvicides.

## 1. Introduction

Beyond the nuisance factor, mosquitoes are carriers, or vectors, for some of humanity’s most deadly illnesses, and they are public enemy number one in the fight against global infectious disease. Mosquito-borne diseases cause millions of deaths worldwide every year with a disproportionate effect on children and the elderly in developing countries [1]. The mosquitoes, *Aedes albopictus* Skuse (*Stegomyia albopictus*), *Anopheles sinensis* Wiedemann and *Culex pipiens pallens* Coquillett (Diptera: Culicidae) are three worldwide insects causing dreadful nuisance and transmitting many harmful diseases [1]. The Asian tiger mosquito (*Ae. albopictus*) and yellow fever mosquito (*Ae. aegypti*) are regarded as the major vectors for transmission of dengue fever in China. Cases of dengue fever and dengue hemorrhagic fever have boosted every year [2]. On the other hand, malaria remains one of the most important diseases worldwide and one of the world’s biggest killers, with 350–500 million cases occurring annually that are transmitted by *Anopheles* sp. mosquitoes. *An. sinensis* is incriminated as the most important vector of malaria and lymphatic filariasis in Southeast Asia. In China, it also was responsible for the transmission of the filalarial parasite (*Wuchereria bancrofti*), and arthropod roundworm (*Romanomermis jingdeensis*) [2,3]. Moreover, the common house mosquito (*C. pipiens pallens*) is the major vector of wuchereriasis and epidemic encephalitis B, which cause millions of deaths every year, especially in India and Africa [3,4,5]. Vector control programs using synthetic insecticides organophosphates (e.g., temephos, fenthion, and malathion), and insect growth regulators (e.g., diflubenzuron, methoprene) have long been utilized to stop the transmission of these diseases. However, frequent and indiscreet application of these synthetic insecticides has caused the disruption of the natural biological control systems and sometimes resulted in the widespread development of resistance as well as undesirable effects on non-target organisms, toxic residues in food, workers’ safety, and high cost of procurement [6,7]. As a result, there is a critical need for the development of alternatives to synthetic insecticides. Essential oils and their constituents have been recommended as alternative sources for insect control, predominantly because some are selective, biodegrade to nontoxic products, and have minimal impacts on non-target organisms and the environment [6]. Many essential oils and constituent compounds that come from various essential oils can put forth toxic activity against mosquito species [8,9,10,11,12,13,14,15]. During our screening project for new agrochemicals from the wild plants and Chinese medicinal herbs, essential oil of *Echinops grijsii* Hance (syn. *Echinops cathayanus* Kitag.) (Family: Asteraceae) roots was found to possess larvicidal activity against the Asian tiger mosquito, *Ae. albopictus* and *An. sinensis* as well as *C. pipiens pallens*.

Eastern China globe thistle (*E. grijsii*) is a perennial herb that is 30–80 cm tall, found in Anhui, Fujian, Guangxi, Henan, Hubei, Jiangsu, Jiangxi, South Liaoning, Shandong, Taiwan, and Zhejiang province, China [16]. The roots of this herb have been used to clear heat, expel miasma and stimulate milk secretion for a long history in traditional Chinese medicine, and it has been recorded in Chinese Pharmacopoeia as one of the sources for Radix Echinopsis [17]. Previous chemical investigation on this plant demonstrated the presence of thiophenes, triterpenoids and neolignan glycosides [18,19,20,21,22,23]. Chemical composition of *E. grijsii* essential oil was also determined [24]. However, a literature survey has shown that there is no report on larvicidal activity of the essential oil of *E. grijsii* roots against mosquitoes, thus we decided to investigate larvicidal activity of the essential oil against the three mosquitoes and isolate any active constituent compounds from the essential oil using bioactivity directed fractionation.

## 2. Results and Discussion

### 2.1. Essential Oil Chemical Composition

The yield of yellow essential oil of *E. grijsii* roots was 0.46% (*v*/*w* based on dry weight) while its density was measured to be 0.92 g/mL. A total of 31 components derived from the essential oil of *E. grijsii* roots were identified, accounting for 98.53% of the crude essential oil. The main constituents of *E. grijsii* essential oil were 5-(3-buten-1-yn-1-yl)-2,2′-bithiophene (5-BBT) (27.63%), α-terthienyl (α-T) (14.95%), 1,8-cineole (5.56%), *cis*-β-ocimene (5.01%), and *cis*-β-farnesene (4.71%) (Table 1). The essential oil of *E. grijsii* roots had higher thiophenes (47.62%) than monoterpenoids (22.63%) and sesquiternoids (25.96%) (Table 1). It had some differences to the essential oil of *E. grijsii* roots measured in the previous report [24]. The essential oil of *E. grijsii* roots collected from Henan province mainly contained *cis*-β-farnesene (25.18%), 5-BBT (19.67%), β-bisabolene (12.11%), α-T (8.36%), and β-bergamotene (4.57%) [24]. This suggests that high content of thiophenes may be a characteristic constituent of the essential oil of *E. grijsii*.

### 2.2. Isolated Bioactive Compounds

**Figure 1:**
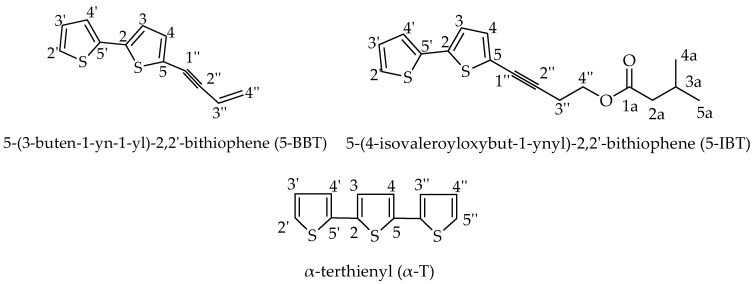
Larvicidal thiophenes isolated from the essential oil of *Echinops grijsii* roots.

Three bioactive constituents, 5-BBT, α-T and 5-(4-isovaleroyloxybut-1-ynyl)-2,2′-bithiophene (5-IBT), were separated by using bioassay-guided fractionation and identified based on their spectroscopic data and comparison with literature vales. Their chemical structures are provided in Figure 1.

### 2.3. Larvicidial Activity

The essential oil of *E. grijsii* roots exhibited larvicidal activity against the fourth instar larvae of *Ae. albopictus*, *An. sinensis* and *C. pipiens pallens* with LC_50_ values of 2.65 μg/mL 3.43 μg/mL and 1.47 μg/mL, respectively (Table 2). The isolated constituents, 5-BBT and 5-IBT possessed sound larvicidal activity against the fourth instar larvae of *Ae. albopictus* with LC_50_ values of 0.34 μg/mL and 0.45 μg/mL, respectively (Table 2). 5-BBT and 5-IBT had LC_50_ values against the fourth instar larvae of *An. sinensis* of 1.36 μg/mL and 5.36 μg/mL respectively. Moreover, 5-BBT, and 5-IBT also possessed LC_50_ values against the fourth instar larvae of *C. pipiens pallens* of 0.12 μg/mL and 0.33 μg/mL, respectively.

In this study, a positive correlation was obtained between the essential oil/isolated constituents’ concentration and the larvicidal activity (*p* < 0.15), the rate of mortality being directly proportional to the concentration, indicating a dose-dependent effect on mortality (Table 2). The essential oil of *E. grijsii* roots exhibited larvicidal activity against the three species of mosquitoes. *Ae. albopictus* and *An. sinensis* larvae were two times (based on the LC_50_ values) more tolerant to toxicity of the essential oil than *C. pipiens pallens* larvae. The essential oil of *E. grijsii* roots displayed stronger larvicidal activity (based on LC_50_ values, no overlaps in 95% confidence limits) than a positive control, rotenone against the fourth instar larvae of *Ae. albopictus* and *C. pipiens pallens* because it had LC_50_ values of 3.75 μg/mL and 1.88 μg/mL against the two species of mosquitoes (Table 2), but was less toxic than rotenone against the fourth instar larvae of *An. sinensis*. Moreover, compared with the other essential oils using the same bioassay in the literature, essential oil of *E. grijsii* exhibited stronger larvicidal activity against *Ae. albopictus* larvae, e.g., essential oils of *Allium macrostemon* (LC_50_ = 72.86 μg/mL) [25] and *Allium tuberosum* (LC_50_ = 18.0 μg/mL) [10], *Eucalyptus urophylla* (LC_50_ = 95.5 μg/mL) and *E. camaldulensis* (LC_50_ = 31.0 μg/mL) [26], *Foeniculum vulgare* (LC_50_ = 142.9 μg/mL) [27], *Saussurea lappa* (LC_50_ = 12.41 μg/mL) [4], *Tetradium glabrifolium* (LC_50_ = 8.2 μg/mL) [11] and *Toddalia asiatica* (LC_50_ = 69.09 μg/mL) [28].

The isolated constituents, 5-BBT and 5-IBT, possessed stronger larvicidal activity (based on LC_50_ values) against the fourth instar larvae of *Ae. albopictus* and *C. pipiens pallens* than a very famous and widely studied thiophene, α-T (LC_50_ = 1.41 μg/mL and 1.38 μg/mL, respectively) (Table 2). Moreover, all the three isolated thiophenes exhibited stronger larvicidal activity (based on LC_50_ values) than the crude essential oil against the two species of mosquitoes. However, when using *An. sinensis* as a target mosquito, 5-IBT demonstrated less larvicidal activity (based on LC_50_ values) than α-T and the crude essential oil (Table 2). Among the three isolated compounds, 5-BBT showed the strongest larvicidal activity against the three species of mosquitoes and was shown to be almost four times more toxic than α-T against the fourth instar larvae of *Ae. albopictus* and 12 times more toxic to the fourth instar larvae of *C. pipiens pallens* (based on the LC_50_ values). Moreover, compared with rotenone (based on the LC_50_ values), 5-BBT was shown to be 11 times and 16 times more toxic to the fourth instar larvae of *Ae. albopictus* and *C. pipiens pallens*, respectively (Table 2) and 5-BBT showed the same level of toxicity against *An. sinensis* as rotenone. It is suggested that larvicidal activity of the essential oil of *E. grijsii* against three species of mosquitoes may be attributed to 5-BBT.

Based on gas chromatography-mass spectrometry (GC-MS) analysis, 5-IBT was not a major constituent in the essential oil and 1,8-cineole and *cis*-β-ocimene were two of the four main compounds identified in the essential oil (Table 1). However, the two compounds were not separated from the essential oil in this study by using bioassay-directed fractionation. Moreover, the two constituents were shown to exhibit weak larvicidal activity against several mosquitoes [15,26,29]. In the previous reports, naturally occurring thiophenes and polyacetylenes have been demonstrated to possess toxic or phototoxic activity against insects, especially larval mosquitoes [30,31,32,33,34,35]. α-T was proven to be phototoxic against a number of mosquito larvae, *Ae. aegypti*, *Ae. albopictus*, *Ae. atropalpus*, *Ae. epactius*, *Ae. intrudens*, *An. gambiae*, *An. stephensi*, *Culex tarsalis*, and *C. tritaeniorhynchus* [36,37,38]. It exhibits unique free radical generating properties and can generate single oxygen. It inhibits several enzymes such as glucose-6-phosphate dehydrogenase, malate dehydrogenase, acetylcholinesterase and superoxide dismutase [39]. 5-BBT was demonstrated to exhibit cytotoxic activity against tumor cells [40], antifungal and antibacterial activities [41,42]. Moreover, 5-BBT was shown to possess insecticidal activities against several herbivorous insects, such as *Ostrinia nubilalis*, *Euxoa messoria* and *Manduca sexta* [43]. It was also demonstrated to be both toxic and a feeding deterrent to Formosan subterranean termite, *Coptotermes formosanustermites* [44].

This is the first time that the larvicidal activity of the two isolated constituents, 5-BBT and 5-IBT, against the three species of mosquitoes has been reported. The above findings suggest that larvicidal activity of the essential oil and the isolated constituent compounds, especially 5-BBT against the three mosquitoes is quite promising. As current commonly used larvicides are synthetic pesticides and these synthetic pesticides are also highly toxic to humans and other non-target organisms, the essential oil and its three isolated constituents show potential to be developed as possible natural larvicides for the control of mosquitoes.

It seems that *E. grijsii* roots are harmless to humans because they have been used in traditional medicine to clear heat and stimulate milk secretion [17]. However, there is no experimental data on the toxicity of the essential oil of *E. grijsii* roots and its two constituents, 5-BBT and 5-IBT to humans, to the best of our knowledge. α-T broke down easily when exposed to sunlight and its half-life is approximately 6 h [36]. Rats are able to metabolize α-T and rapidly excrete the metabolites and at least two novel acidic metabolites have been determined [45]. Pure α-T appears to show measurable intraperitoneal toxicity (LD_50_ = 110 mg/kg) in rats, but its “ready to use” formulation (0.1% active ingredient) appears to be nontoxic at the highest dose administrated orally to rats in a pilot toxicity experiment [45]. Thus, to build a practical application for the essential oil and its constituents as novel insecticides, further exploration into the safety of the essential oil/compounds to humans is required. Additional studies on the development of formulations are also necessary to upgrade the efficacy and stability and to cut down cost. Moreover, field evaluation and further investigations on the effects of the essential oil and its constituent compounds on non-target organisms are obligatory.

## 3. Experimental

### 3.1. General

^1^H and ^13^C-NMR spectra were measured on Bruker ACF300 [300 MHz (^1^H)] and AMX500 [500 MHz (^1^H)] instruments (Billerica, MA, USA) using CDCl_3_ as the solvent with tetramethylsilane (TMS) as the internal standard.

### 3.2. Plant Material and Essential Oil

The dried roots of *E. grijsii* (10 kg, harvested from Nanjing, Jiangsu Province, China) were acquired from Anguo Herb Market (Anguo, Hebei Province, China). The sample was chopped into small pieces and soaked in water at a ratio of 1:4 (*w*/*v*) for 1 h, prior to hydrodistillation using a round bottom flask over a period of 6 h. The essential oil was gathered in a specific receiver, determined, dried over anhydrous sulfate, and saved in airtight containers. The species was acknowledged by Dr. Liu, Q.R., College of Life Sciences, Beijing Normal University, Beijing 100875 and a voucher specimen of *E. grijsii* (Compositae-HuadongLuluo-Jiangsu-09) was deposited at the museum of Department of Entomology, China Agricultural University.

### 3.3. Gas Chromatography-Mass Spectrometry

Constituents of the essential oil of *E. grijsii* roots were analyzed by GC-MS using an Agilent 6890N gas chromatograph connected to an Agilent 5973N mass selective detector (Agilent Technologies, Santa Clara, CA 95051, USA). The GC was coupled to a flame ionization detector and a 5% phenyl methyl siloxane (HP-5MS) (30 m × 0.25 mm × 0.25 μm) capillary column (Agilent Technologies). The GC settings were as follows: the early oven temperature was detained at 60 °C for 1 min and raised at 10 °C·min^−1^ to 180 °C, remained at 180 °C for 1 min, and then climbed up at 20 °C·min^−1^ to 280 °C for 15 min. The injector temperature was kept at 270 °C. The samples (1 μL, 1:100, *v*/*v*, in acetone) were instilled, with a split ratio of 1:10. The carrier gas was helium at a flow rate of 1.0 mL·min^−1^. Spectra were scanned from 20 to 550 *m*/*z* at two scans per second. Identification of most constituents was done by comparing their retention indices with those of the literature or with those of authentic compounds available in our laboratories. The retention indices were measured in relation to a homologous series of *n*-alkanes (C8–C24) run under the same operating conditions. Further identification was made by comparing the NIST 05 (Standard Reference Data, Gaithersburg, MD 20899, USA) and Wiley 275 libraries (Wiley, New York) data of the peaks with those reported in literature, mass spectra of the peaks with literature data [46]. Component relative percentages were estimated based on GC peak areas.

### 3.4. Bioassay-Directed Fractionation

The crude essential oil of *E. grijsii* roots (10 mL) was separated on a silica gel (Merck 9385, 1000 *g*, Merck, Darmstadt, Germany) column (85 mm i.d., 850 mm length) by gradient elution with a mixture of solvents (*n*-hexane/ethyl acetate, from 100:0, 100:1, 100:2, ···, to 0:100). Fractions of 500 mL each were gathered and distillated at 40 °C; related fractions according to their thin layer chromatography (TLC) profiles were merged to produce 13 fractions. The screening experiments were done by using 500 ppm solution (firstly diluted in acetone) of various fractions. The larvicidal activity bioassay was made as illustrated above. Fractions (F9, F11–F12) that possessed larvicidal activity, with similar TLC profiles, were put together and further separated by preparative silica gel column chromatography (PTLC) (pre-coated GF254 plate, Qingdao Marine Chemical Plant, Qingdao, China) until the obtainment of the pure compound, 5-(3-buten-1-yn-1-yl)-2,2′-bithiophene (5-BBT, 81 mg), 5-(4-Isovaleroyloxybut-1-ynyl)-2,2′-bithiophene (5-IBT, 77 mg) and α-terthienyl (α-T, 45 mg). The structure of the constituents was elucidated based on nuclear magnetic resonance. NMR spectra of the three isolated compounds are available in the supplementary materials online.

### 3.5. Insects

Mosquito eggs of *Ae. albopictus*, *An. sinensis* and egg masses of *C. pipiens pallens* utilized in bioassays were obtained from a laboratory colony reared in the Department of Vector Biology and Control, Institute for Infectious Disease Control and Prevention, Chinese Center for Disease Control and Prevention. The original eggs of *Ae. albopictus* and *An. sinensis* as well as egg masses of *C. pipiens pallens* were collected from Nanjing, Jiangsu province, China in 1997. Adults were maintained in a cage (60 cm × 30 cm × 30 cm) at 28–30 °C and 75%–85% relative humidity (RH). The females were nourished with blood (Beijing Laboratory Animal Research Center, Beiyuan Road, Beijng, China) every alternate day whereas the males were provided with 10% glucose solution soaked on cotton pad, which were hung in the middle of the cage. A beaker with strips of moistened filter paper was provided for oviposition of *Ae. albopictus*. The eggs laid on paper strips were held wet for 24 h and then dehydrated at room temperature. The dehydrated eggs were put into a plastic tray containing tap water in our laboratory at 26–28 °C and natural summer photoperiod for hatching and yeast pellets served as food for the emerging larvae. Larvae were daily observed until they accomplished the fourth instar, when they were employed for bioassays (within 12 h). The culture procedure of *An. sinensis* and *C. pipiens pallens* is almost the same as that of *Ae. albopictus*. *An. sinensis* deposited in moistened filter paper and *C. pipiens pallens* deposited in tap water and the egg masses or eggs were transferred to a white porcelain basin containing tap water for hatching.

### 3.6. Larvicidal Activity Bioassays

Range-finding studies were made to measure the suitable testing concentrations. The larval mortality bioassays were performed in accordance with the test method of larval susceptibility as recommended by the World Health Organization (WHO) [47]. Each tested compound/oil was diluted/dissolved at first in ethanol to obtain a stock solution (1000 μg/mL) and gradient diluted with distilled water (0.25% Tween 40, purchased from Aladdin Industrial Corporation, Shanghai, China, by volume) as a test solution. Twenty larvae were put into a glass beaker with 250 mL of aqueous suspension of tested material at various concentrations. Five replicates were done concurrently per concentration and with each experiment, a set of control using 0.25% Tween 40 and untreated sets of larvae in distilled water, were also run for comparison. Commercial rotenone (bought from Aladdin Industrial Inc., Shanghai, China) was taken as a positive control. The toxicity of rotenone was measured at 10.00, 5.00, 2.50, 1.25, and 0.63 μg/mL (prepared by the same method as described above). The assays were placed in a growth chamber (12:12 h light:dark (L:D), 26–28 °C, 78%–80% RH). Mortality was recorded after 24 h of exposure.

### 3.7. Isolated Constituent Compounds

*5-(3-Buten-1-ynyl)-2,2′-bithiophene* (Figure 1). Yellowish oil. ^1^H-NMR (CDCl_3,_ 500 MHz) δ (ppm): 5.58 (1 H, d, H-4′′), 5.77 (1 H, d, H-4′′), 6.06 (1 H, dd, H-3′′), 7.04–7.06 (2 H, m, H-3, H-3′), 7.12 (1 H, m, H-4), 7.20 (1 H, m, H-4′), 7.26 (1 H, m, H-2′); ^13^C-NMR (CDCl_3_, 125 MHz) δ (ppm): 138.99 (C-2), 136.69 (C-5′), 132.81 (C-4), 127.92 (C-3′), 126.96 (C-4′′), 125.00 (C-2′), 124.24 (C-4’),123.51 (C-3), 121.77 (C-5), 116.81 (C-3′′), 92.94 (C-2′′), 83.21 (C-1′′). The spectral data matched with the previous reports [18,48].

*5-(4-Isovaleroyloxybut-1-ynyl)-2,2′-bithiophene* (Figure 1). Greenish oil. ^1^H-NMR (CDCl_3,_ 500 MHz) δ (ppm): 0.97 (6 H, d, H-4a, H-5a), 2.12 (H-1, m, H-3a), 2.22 (2 H, m, H-2a), 2.78 (2 H, t, H-3′′), 4.24 (2 H, t, H-4′′), 7.02–7.04 (2 H, m, H-3, H-3′), 7.06 (1 H, d, H-4), 7.18 (1 H, d, H-4′), 7.25 (1 H, d, H-2′); ^13^C-NMR (CDCl_3_, 125 MHz) δ (ppm): 172.24 (C-1a), 138.59 (C-2), 136.69 (C-5′), 132.51 (C-4), 127.92 (C-3′), 124.70 (C-2′), 124.24 (C-4′), 122.51 (C-3), 121.57 (C-5), 90.54 (C-2′′), 75.31 (C-1′′), 61.76 (C-4′′), 20.41 (C-3′′), 43.22 (C-2a), 25.66 (C-3a), 22.3 (C-4a, C-5a). The spectral data matched with the previous reports [18,48].

*α-Terthienyl* (Figure 1). Colorless solid. ^1^H-NMR (CDCl_3,_ 500 MHz) δ (ppm): 7.25 (d, 2 H, H-2′, H-5′′), 7.21 (d, 2 H, H-4′, H-3′′), 7.12 (m, 2 H, H-3′, H-4′), 7.06 (t, 2 H, H-3, H-4); ^13^C-NMR (CDCl_3_, 125 MHz) δ(ppm): 137.10 (C-2, C-5), 136.19 (C-2′′, C-5′), 127.84 (C-2′, C-5′′), 124.44 (C-3, C-4), 124.28 (C-4′, C-3′′), 123.67 (C-3′, C-4′′). The spectral data matched with a previous report [48].

### 3.8. Data Analysis

The observed mortality data were amended for control mortality using Abbott’s formula [49]. The results from all replicates in larvicidal bioassay were subjected to Probit analysis using PriProbit Program V1.6.3 to estimated LC_50_ and LC_90_ values [50] (www.ars.usda.gov/pacific-west-area/parlier/sjvasc/cpq/docs/priprobit-download/). Significant differences in LC_50_ and LC_90_ were based on nonoverlap of the 95% confidence intervals. Regression analysis was adopted to investigate the linear relationship between concentrations and the mean value of larval mortalities.

## 4. Conclusions

The present work indicated that the essential oil of *E. grijsii* roots reveals strong larvicidal activity against larval mosquitoes, *Ae. albopictus*, *An. sinensis* and *C. pipiens pallens*. The isolated three constituents, exhibited stronger larvicidal activity than the commercial insecticide, rotenone. Our results suggested that the essential oil of *E. grijsii* roots and the three isolated constituents, especially 5-BBT, may be recommended as being effective in a mosquito control program but need to be further appraised for safety in humans and to improve their activity.

## Figures and Tables

**Table 1:** Constituents identified from the essential oil of *Echinops grijsii* roots.

Peak No	Compound	RRI ^a^	RI ^b^	Percent Composition
	Monoterpenoids			22.63
1	α-Pinene *	939	939	0.89
2	β-Pinene *	978	980	3.92
3	β-Myrcene *	990	991	1.94
4	1,8-Cineole *	1031	1033	5.56
5	*cis*-β-Ocimene	1038	1037	5.01
6	Artemisia ketone	1064	1062	0.89
7	Linalool *	1098	1097	1.54
8	Carvone *	1243	1242	2.88
	Sesquiterpenoids			25.96
9	β-Cubebene	1388	1388	0.97
10	β-Maaliene	1411	1411	0.29
11	Caryophyllene *	1420	1418	3.84
12	α-Santalene	1424	1424	0.52
13	*cis*-Thujopsene	1427	1429	0.96
14	α-Bergamotene	1433	1436	0.42
15	α-Caryophyllene	1453	1455	0.84
16	*cis*-β-Farnesene	1458	1457	4.71
17	*cis*-β-Guaiene	1485	1488	0.50
18	α-Selinene	1494	1493	1.66
19	Virdiflorene	1497	1497	1.06
20	α-Bulnesene	1505	1505	0.95
21	β-Bisabolene	1506	1509	0.46
22	δ-Cadinene	1524	1524	2.32
23	Spathulenol	1578	1576	1.56
24	Caryophyllene oxide *	1583	1581	3.53
25	τ-Cadinol	1642	1640	0.25
26	*trans*-α-Bergamotol	1714	-	1.12
	Thiophenes			47.62
27	5-(3-Buten-1-yn-1-yl)-2,2’-bithiophene	1941	1935	27.63
28	5-(4-Isovaleroyloxybut-1-ynyl)-2,2’-bithiophene	2062	-	2.34
29	α-Terthienyl	2243	2240	14.95
	Others			2.32
30	Eugenol *	1356	1356	1.54
31	Methyleugenol *	1403	1401	0.78
	Total identified			98.53

^a^ RRI, retention index as determined on a 5% phenyl methyl siloxane (HP-5MS) column using the homologous series of *n*-hydrocarbons; ^b^ RI, literature retention indices on the HP-5MS column; * Identification by co-injection of authentic compounds.

**Table 2:** Larvicidal activity of the essential oil of *Echinops grijsii* roots and the isolated constituents against fourth-instar larvae of *Aedes albopictus*, *Anopheles sinensis* and *Culex pipiens pallens*.

Insects	Treatment *	LC_50_ (μg/mL)	LC_95_ (μg/mL)	Slope ± SD	χ^2^	*p*
		(95% CL)	(95% CL)			
*Aedes albopictus*	Essential oil	2.65	4.65	5.23 ± 0.52	17.79	0.0001
		(2.54–2.71)	(4.17–5.42)			
	5-BBT	0.34	0.72	4.63 ± 0.43	8.05	0.0179
		(0.29–0.39)	(0.66–0.81)			
	5-IBT	0.45	0.66	7.67 ± 0.66	7.84	0.0214
		(0.38–0.49)	(0.61–0.75)			
	α-T	1.41	2.19	6.72 ± 0.61	7.71	0.0212
		(1.33–1.60)	(1.89–2.41)			
	Rotenone	3.75	9.45	4.12 ± 0.65	9.11	0.0001
		(3.55–3.98)	(8.65–10.32)			
*Anopheles sinensis*	Essential oil	3.43	5.67	6.78 ± 0.63	11.09	0.0089
		(3.11–3.69)	(5.04–6.21)			
	5-BBT	1.36	1.93	8.47 ± 0.67	12.03	0.0121
		(1.27–1.45)	(1.75–2.09)			
	5-IBT	5.36	11.26	3.97 ± 0.37	13.64	0.0219
		(4.64–6.09)	(10.21–12.05)			
	α-T	1.79	2.54	8.38 ± 0.76	12.19	0.0154
		(1.67–1.91)	(2.36–2.81)			
	Rotenone	1.25	2.24	2.37 ± 0.18	16.21	0.0033
		(1.07–1.33)	(2.01–2.45)			
*Culex pipiens pallens*	Essential oil	1.47	2.21	7.18 ± 0.67	5.71	0.0577
		(1.34–1.52)	(1.99–2.48)			
	5-BBT	0.12	0.18	6.64 ± 0.59	8.69	0.0182
		(0.09–0.14)	(0.16–0.21)			
	5-IBT	0.33	0.54	5.81 ± 0.56	13.41	0.0129
		(0.26–0.41)	(0.47–0.63)			
	α-T	1.38	2.15	6.65 ± 0.66	13.29	0.0193
		(1.69–1.49)	(1.88–2.35)			
	Rotenone	1.88	3.74	5.33 ± 0.51	11.67	0.0044
		(1.63–1.93)	(3.51–4.03)			

* 5-BBT: 5-(3-buten-1-yn-1-yl)-2,2′-bithiophene; 5-IBT: 5-(4-isovaleroyloxybut-1-ynyl)-2,2′-bithiophene; α-T: terthienyl; CL: confidence limits, SD: standard deviation; *p*: probability; χ^2^: Chi-square value.

## Supplementary Materials

The supplementary materials are available online at www.mdpi.com/1420-3049/22/2/205/s1. Figure S1: Flower (a) and roots (b) of *Echinops grijsii.* Figure S2: ^1^H-NMR and ^13^C-NMR spectra of 5-(3-buten-1-yn-1-yl)-2,2′-bithiophene (5-BBT). Figure S3: ^1^H-NMR and ^13^C-NMR spectra of 5-(4-isovaleroyloxybut-1-ynyl)-2,2′-bithiophene (5-IBT). Figure S4: ^1^H-NMR and ^13^C-NMR spectra of α-terthienyl (*α-*T).

## References

[1] Cui F., Raymond M., Qiao C.L. (2006). Insecticide resistance in vector mosquitoes in China. Pest Manag. Sci..

[2] Chang X., Zhong D., Fang Q., Hartsel J., Zhou G., Shi L., Fang F., Zhu C., Yan G., Mutuku F. (2014). Multiple resistances and complex mechanisms of *Anopheles sinensis* mosquito: A major obstacle to mosquito-borne diseases control and elimination in China. PLoS Negl. Trop. Dis..

[3] Liu Z.L., Liu Q.Z., Du S.S., Deng Z.W. (2012). Mosquito larvicidal activity of alkaloids and limonoids derived from *Evodia rutaecarpa* unripe fruits against *Aedes albopictus* (Diptera: Culicidae). Parasitol. Res..

[4] Liu Z.L., He Q., Chu S.S., Wang C.F., Du S.S., Deng Z.W. (2012). Essential oil composition and larvicidal activity of *Saussurea lappa* roots against the mosquito *Aedes albopictus* (Diptera: Culicidae). Parasitol. Res..

[5] Wang Z.Q., Perumalsamy H., Wang M., Shu S.H., Ahn Y.J. (2016). Larvicidal activity of *Magnolia denudata* seed hydrodistillate constituents and related compounds and liquid formulations towards two susceptible and two wild mosquito species. Pest Manag. Sci..

[6] Isman M.B. (2006). Botanical insecticides, deterrents, and repellents in modern agriculture and an increasingly regulated world. Annu. Rev. Entomol..

[7] Regnault-Roger C., Vincent C., Arnason J.T. (2012). Essential oils in insect control: Low-risk products in a high-stakes world. Annu. Rev. Entomol..

[8] Chang K.S., Shin E.H., Yoo D.H., Ahn Y.J. (2014). Enhanced toxicity of binary mixtures of *Bacillus thuringiensis* subsp. *israelensis* and three essential oil major constituents to wild *Anopheles sinensis* (Diptera: Culicidae) and *Aedes albopictus* (Diptera: Culicidae). J. Med. Entomol..

[9] Liang Y.P., Li X.W., Gu Z.M., Qin P.W., Ji M.S. (2015). Toxicity of amorphigenin from the seeds of *Amorphafruticosa* against the larvae of *Culexpipienspallens* (Diptera: Culicidae). Molecules.

[10] Liu X.C., Zhou L., Liu Q., Liu Z.L. (2015). Laboratory evaluation of larvicidal activity of the essential oil of *Allium tuberosum* roots and its selected major constituent compounds against *Aedes albopictus*. J. Med. Entomol..

[11] Liu X.C., Liu Q., Chen X.B., Zhou L., Liu Z.L. (2015). Mosquito larvicidal constituents from the essential oil of *Tetradium glabrifolium* fruits against *Aedes albopictus*. Pest Manag. Sci..

[12] Zhu S.L., Liu X.C., Liu Z.L., Xu X.F. (2015). Chemical composition of *Salvia plebeian* R. Br. essential oil and its larvicidal activity against *Aedesaegypti* L. Trop. J. Pharm. Res..

[13] Liu X.C., Liu Q., Chen X.B., Zhou L., Liu Z.L. (2015). Larvicidal activity of the essential oil of *Youngia japonica* aerial parts and its constituents against *Aedes albopictus*. Z. Naturforsch..

[14] Liu X.C., Liu Q., Zhou L., Liu Z.L. (2015). Larvicidal activity of essential oil derived from *Illicium henryi* Diels (Illiciaceae) leaf. Trop. J. Pharm. Res..

[15] Liu Y., Liu X.C., Liu Q., Niu C., Liu Z.L. (2015). Evaluation of larvicidal activity of the essential oil of *Illicium difengpi* and its major constituents against the *Aedes aegypti* mosquito. Trop. J. Pharm. Res..

[16] Wu Z.Y., Peter H.R., Hong D.Y. (2009). Flora of China.

[17] National Pharmacopoeia Committee (2005). Pharmacopoeia of the People’s Republic of China.

[18] Guo D.A., Cui Y.J., Lou Z.C., Gao Y., Huang L.R. (1992). Chemical constituents of east China globe thistle (*Echinops grijisii*). Chin. Tradit. Herb. Drug.

[19] Lin Y.L., Huang R.L., Kuo Y.H., Chen C.F. (1999). Thiophenes from *Echinops grijsii* Hance. Chin. Pharm. J..

[20] Koike K., Jia Z.H., Guo H.Z., Nikaido T., Liu Y., Zhao Y.Y., Guo D.A. (2002). A new neolignan glycoside from the roots of *Echinops grijissii*. Nat. Med..

[21] Liu Y., Ye M., Guo H.Z., Zhao Y.Y., Guo D.A. (2002). New thiophenes from *Echinops grijisii*. J. Asian Nat. Prod. Res..

[22] Zhang P., Jin W.R., Shi Q., He H., Ma Z.J., Qu H.B. (2008). Two novel thiophenes from *Echinops grijissi* Hance. J. Asian Nat. Prod. Res..

[23] Zhang P., Liang D., Jin W.R., Qu H.B., Cheng Y.Y., Li X., Ma Z.J. (2009). Cytotoxic thiophenes from the root of *Echinops grijisii* Hance. Z. Naturforsch..

[24] Guo D.A., Lou Z.C., Liu Z.A. (1994). Chemical components of volatile oil from *Echinops grijsii* Hance. Chin. J. Chin. Mater. Med..

[25] Liu X.C., Liu Q., Zhou L., Liu Z.L. (2014). Evaluation of larvicidal activity of the essential oil of *Allium macrostemon* Bunge and its selected major constituent compounds against *Aedes albopictus* (Diptera: Culicidae). Parasite Vector.

[26] Cheng S.S., Huang C.G., Chen Y.J., Yu J.J., Chen W.J., Chang S.T. (2009). Chemical compositions and larvicidal activities of leaf essential oils from two eucalyptus species. Bioresour. Technol..

[27] Conti B., Canale A., Bertoli A., Gozzini F., Pistelli L. (2010). Essential oil composition and larvicidal activity of six Mediterranean aromatic plants against the mosquito *Aedes albopictus* (Diptera: Culicidae). Parasitol. Res..

[28] Liu X.C., Dong H.W., Zhou L., Du S.S., Liu Z.L. (2012). Essential oil composition and larvicidal activity of *Toddalia asiatica* roots against the mosquito *Aedes albopictus* (Diptera: Culicidae). Parasitol. Res..

[29] Santos G.K.N., Dutra K.A., Lira C.S., Lima B.N., Napoleão T.H., Paiva P.M.G., Maranhão C.A., Brandão S.S.F., Navarro D.M.A.F. (2014). Effects of *Croton rhamnifolioides* essential oil on oviposition, larval toxicity and trypsin activity. Molecules.

[30] Arnason J.T., Philogene B.J.R., Morand P., Imrie K., Iyengar S., Duval F., Soucy-Breau C., Scaiano J.C., Werstiuk N.H., Hasspieler B. (1989). Naturally occurring and synthetic thiophenes as photoactivated insecticides. ACS Symp. Ser..

[31] Marles R.J., Compadre R.L., Compadre C.M., Soucy-Breau C., Redmond R.W., Duval F., Mehta B., Morand P., Scaiano J.C., Arnason J.T. (1991). Thiophenes as mosquito larvicides: Structure-toxicity relationship analysis. Pestic. Biochem. Physiol..

[32] Tian Y.Q., Wei X.Y., Xu H.H. (2006). Photoactivated insecticidal thiophene derivatives from *Xanthopappus subacaulis*. J. Nat. Prod..

[33] Arnason J.T., Philogene B.J.R., Berg C., MacEachern A., Kaminski J., Leitch L.C., Morand P., Lam J. (1986). Phototoxicity of naturally occurring and synthetic thiophene and acetylenean alogues to mosquito larvae. Phytochemistry.

[34] Arnason J.T., Swain T., Wat C.K., Graham E.A., Partington S., Towers G.H.N., Lam J. (1991). Mosquito larvicidal activity of polyacetylenes from species in the Asteraceae. Biochem. Syst. Ecol..

[35] Nakano H., Ali A., Rehman J.U., Mamonov L.K., Cantrell C.L., Khan I.A. (2014). Toxicity of thiophenes from *Echinops transiliensis* (Asteraceae) against *Aedes aegypti* (Diptera: Culicidae) larvae. Chem. Biodivers..

[36] Philogene B.J., Arnason J.T., Berg C.W., Duval F., Champagne D., Taylor R.G., Leitch L.C., Morand P. (1985). Synthesis and evaluation of the naturally occurring phototoxin, α-terthienyl, as a control agent for larvae of *Aedes intrudens*, *Aedes atropalpus* (Diptera: Culicidae) and *Simulium verecundum* (Diptera: Simuliidae). J. Econ. Entomol..

[37] Kagan J., Kagan E., Patel S., Perrine D., Bindokas V. (1987). Light-dependent effects of α-terthienyl in eggs, larvae, and pupae of mosquito *Aedes aegypti*. J. Chem. Ecol..

[38] Zhang L.M., Zhang Q., Lv H.F., Zhu P.X. (2005). Toxicity of α-terthienyl to larvae of deltamethrin- resistant strains of *Aedes albopictus*. J. Jinan Univ..

[39] Nivsarkar M., Cherian B., Padh H. (2001). α-Terthienyl: A plant-derived new generation insecticide. Curr. Sci..

[40] Jin W.R., Shi Q., Hong C.T., Cheng Y.Y., Ma Z.J., Qu H.B. (2008). Cytotoxic properties of thiophenes from *Echinops grijissi* Hance. Phytomedicine.

[41] Fokialakis N., Cantrell C.L., Duke S.O., Skaltsounis A.L., Wedge D.E. (2006). Antifungal activity of thiophenes from *Echinops ritro*. J. Agric. Food Chem..

[42] Campos B.M.M.G., Cirio A.T., Galindo V.M.R., Aranda R.S., Torres N.W., Perez-Lopez L.A. (2011). Activity against *Streptococcus pneumoniae* of the essential oil and 5-(3-buten-1-ynyl)-2,2′-bithienyl isolated from *Chrysactinia mexicana* roots. Nat. Prod. Commun..

[43] Champagne D.E., Arnason J.T., Philogene B.J.R., Morand P., Lam J. (1986). Light-mediated allelochemical effects of naturally occurring polyacetylenes and thiophenes from Asteraceae on herbivorous insects. J. Chem. Ecol..

[44] Fokialakis N., Osbrink W.L.A., Mamonov L.K., Gemejieva N.G., Mims A.B., Skaltsounis A.L., Lax A.R., Cantrell C.L. (2006). Antifeedant and toxicity effects of thiophenes from four *Echinops* species against the formosan subterranean termite, *Coptotermes formosanus*. Pest Manag. Sci..

[45] Marles R., Durst T., Kobaisy M., Soucy-Breau C., Abou-Zaid M., Arnason J.T., Kacew S., Kanjanapothi D., Rujjanawate C., Meckes M. (1995). Pharmacokinetics, metabolism and toxicity of the plant-derived phototoxin α-terthienyl. Pharmacol. Toxicol..

[46] Adams R.P. (2007). Identification of Essential Oil Components by Gas Chromatography/Mass Spectrometry.

[47] World Health Organization (1996). Report of the WHO Informal Consultation, on the Evaluation and Testing of Insecticides.

[48] Wang Y., Li X., Meng D.L., Li Z.L., Zhang P., Xu J. (2006). Thiophenes from *Echinops latifolius*. J. Asian Nat. Prod. Res..

[49] Abbott W.S. (1925). A method of computing the effectiveness of an insecticide. J. Econ. Entomol..

[50] Sakuma M. (1998). Probit analysis of preference data. Appl. Entomol. Zool..

