# Supplementary material for: Identification of Larvicidal Constituents of the Essential Oil of Echinops grijsii Roots against the Three Species of Mosquitoes
Source: Molecules. 2017 Jan 27;22(2):205. doi: 10.3390/molecules22020205 (PMC6155871; doi:10.3390/molecules22020205)
Supplement: Supplementary file 1 [file molecules-22-00205-s001.pdf]

Supplementary Materials: Identification of Larvicidal Constituents of the Essential Oil of *Echinops grijsii* Roots against the Three Species of Mosquitoes

Mei Ping Zhao, Qi Zhi Liu, Qiyong Liu and Zhi Long Liu

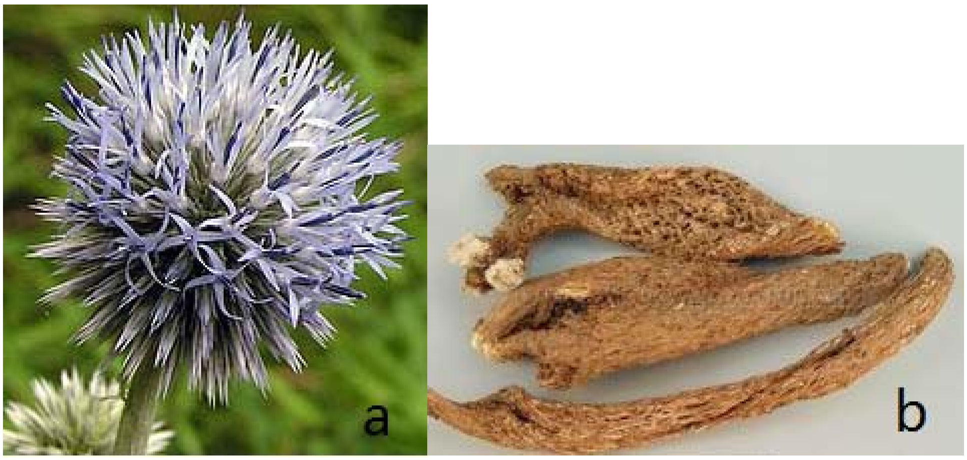

Figure S1. Flower (a) and roots (b) of *Echinops grijsii.*

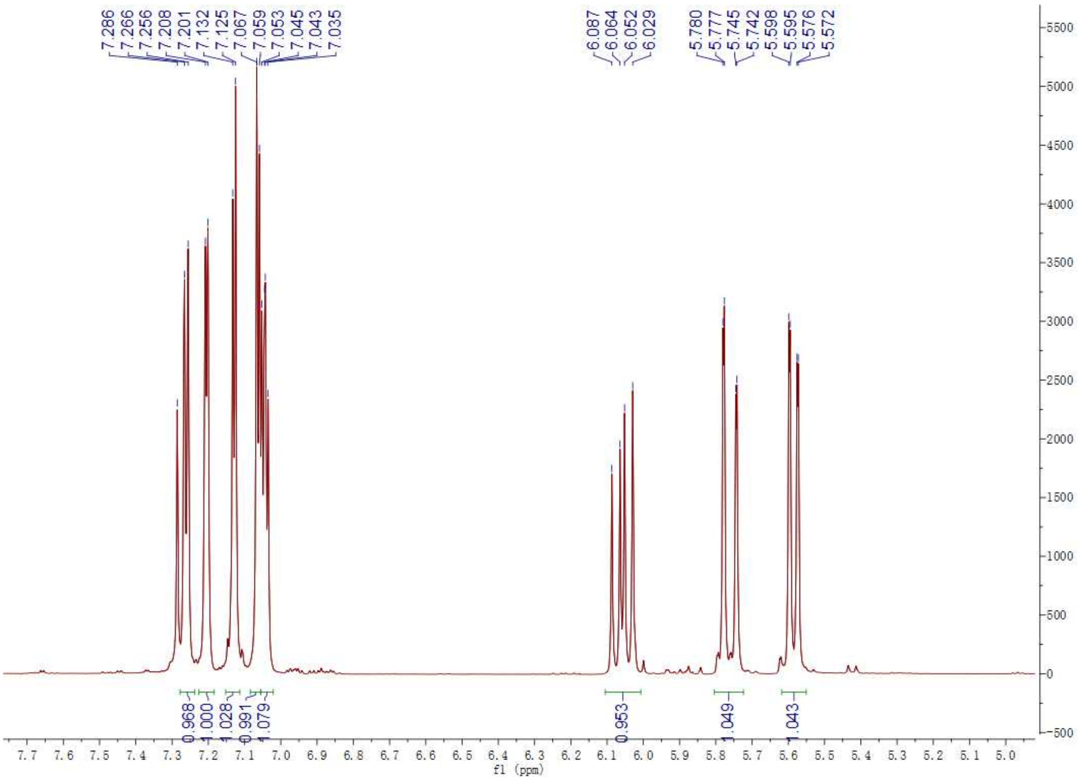

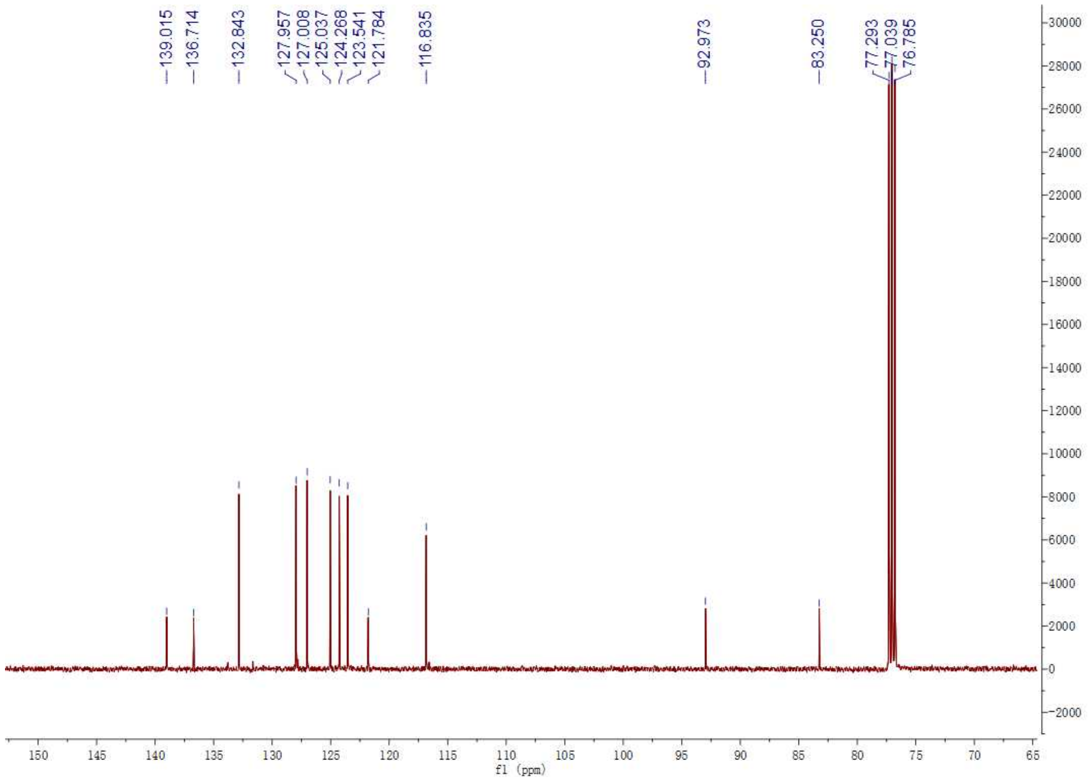

Figure S2. ^{1}H-NMR and ^{13}C-NMR spectra of 5-(3-buten-1-yn-1-yl)-2,2′-bithiophene (5-BBT).

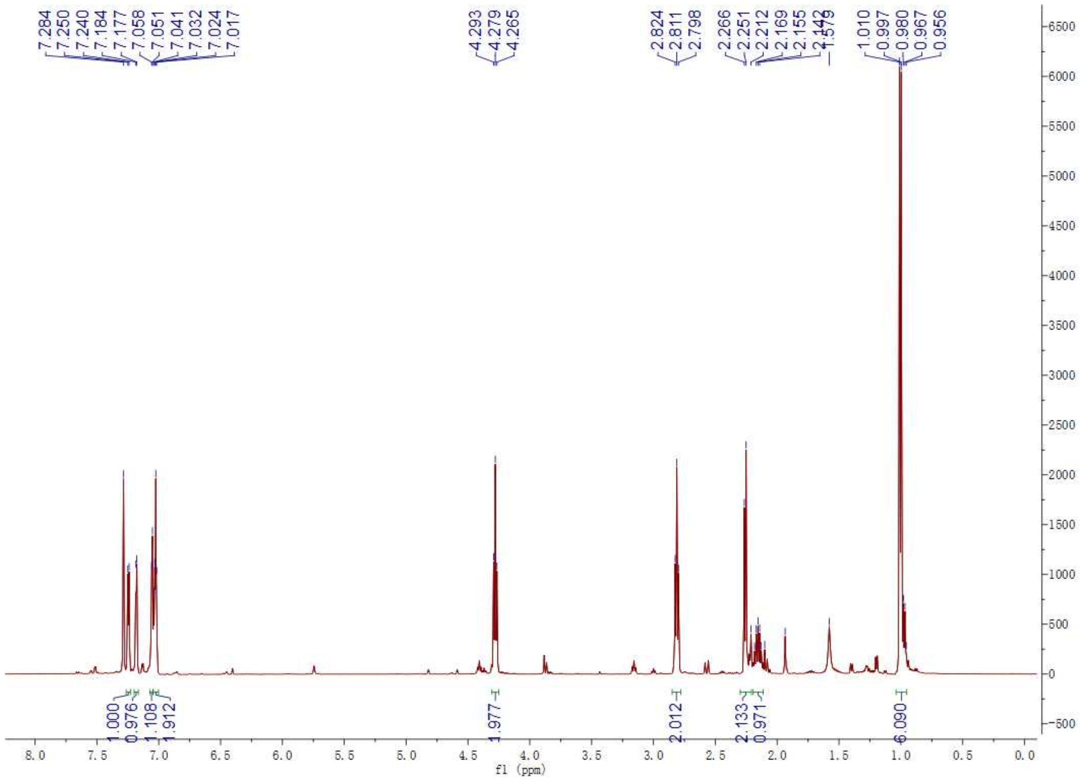

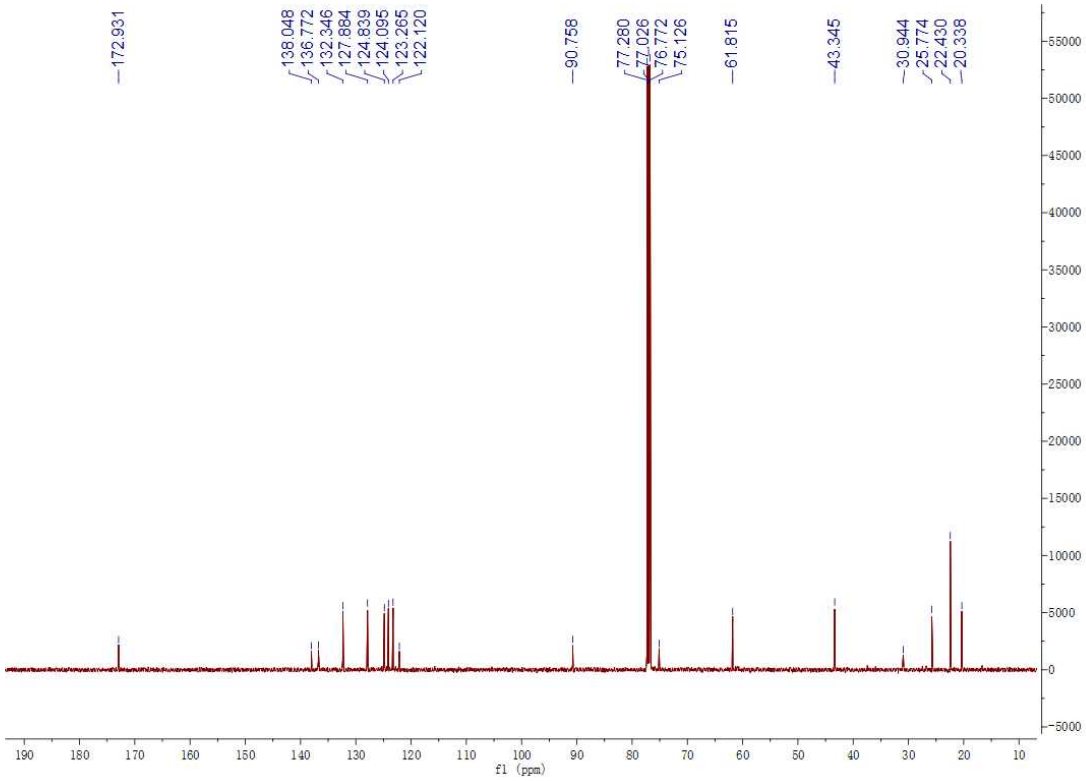

Figure S3. ^{1}H-NMR and ^{13}C-NMR spectra of 5-(4-isovaleroyloxybut-1-ynyl)-2,2′-bithiophene (5-IBT).

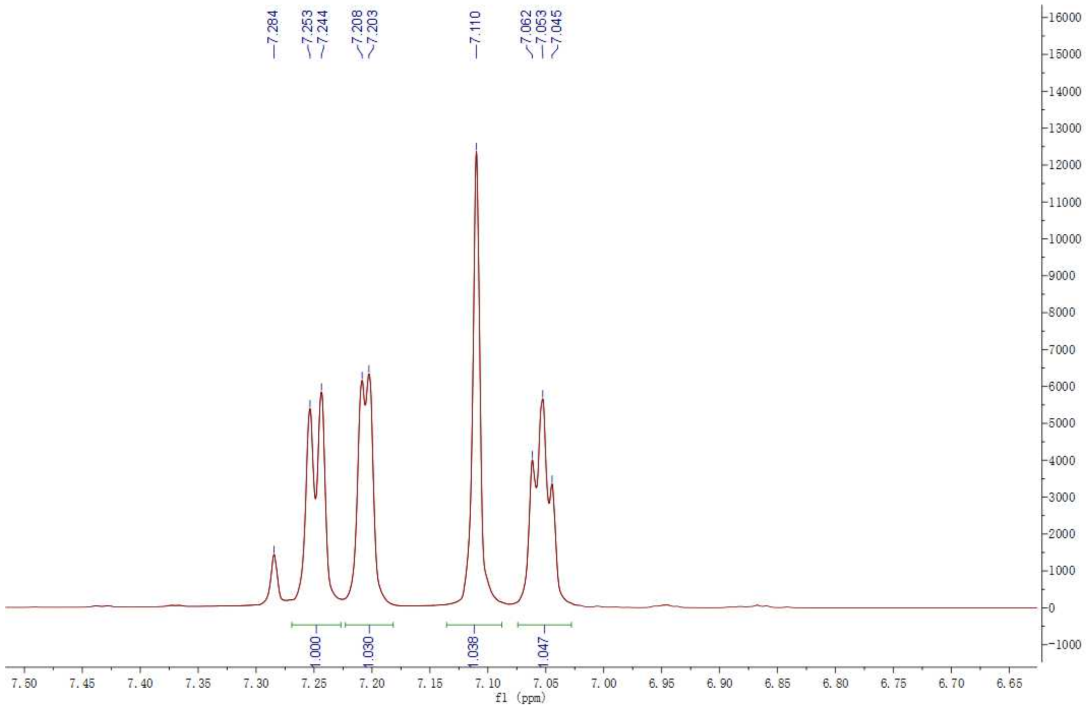

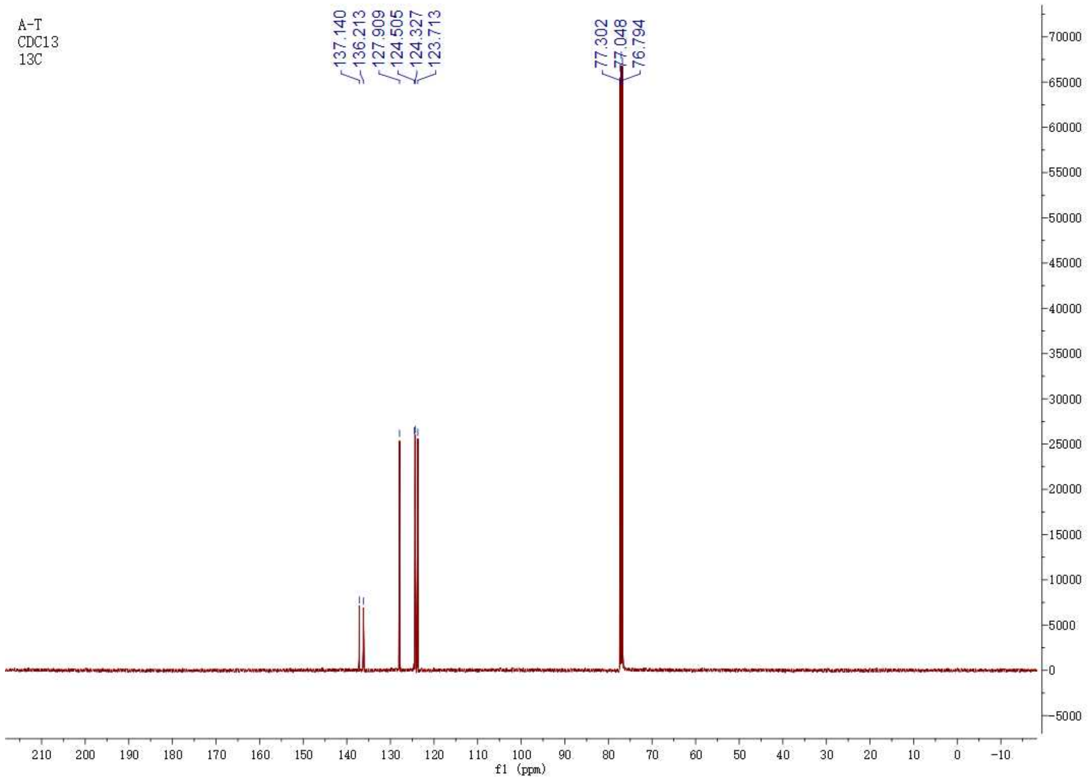

Figure S4. ^{1}H-NMR and ^{13}C-NMR spectra of α-terthienyl (α-T).
